# Editorial: Natural polyphenols and metabolic syndrome

**DOI:** 10.3389/fnut.2023.1190577

**Published:** 2023-04-20

**Authors:** Juntao Zhang, Jianjun Deng, Haixia Yang

**Affiliations:** ^1^College of Food Science and Nutritional Engineering, China Agricultural University, Beijing, China; ^2^State Key Laboratory of Vegetable Biobreeding, Institute of Vegetables and Flowers, Chinese Academy of Agricultural Sciences, Beijing, China

**Keywords:** polyphenols, obesity, inflammation, lipid metabolism, diabetes

Metabolic syndrome is a chronic disease characterized by hypertension, obesity, diabetes, and hyperlipidemia, as defined by the World Health Organization (1). Global epidemiological surveys demonstrate that the current incidence rate of metabolic syndrome is between 20 and 45% (2), and is predicted to increase to about 53% by 2035 (3). Multiple risk factors, including an unhealthy diet, inactivity, and environmental factors, increase the chances of developing metabolic syndrome. Therefore, intervention does not only involve exploring clinical therapeutic treatment at the point of diagnosis, but it is also crucial to prevent the occurrence of metabolic syndrome and its complications by keeping a healthy lifestyle, such as by doing sports or by adding vegetables, low sugar fruit, and less salt to the diet.

Natural polyphenols, common specialized metabolites from plants, are a class of bioactive compounds widely found in fruits, vegetables, tea, and herbal medicines. They confer potentially beneficial activity, by regulating gut microbiota and enacting anti-inflammation or antioxidation (4–6). A diet rich in polyphenols alleviates the symptoms and risk factors of metabolic syndrome, such as by reducing blood pressure, improving insulin-resistance and modulating lipid metabolism (7, 8). In this Research Topic on “*Natural polyphenols and metabolic syndrome*,” a series of original papers were published that explore the knowledge of natural polyphenols related to their composition, chemical characterization, and their ability to alleviate obesity and diabetes by modulating gut microbiota, conferring antioxidation, and improving metabolism, and immunity.

Traditional plants are often used as a source of functional food or pharmaceutical in the industry because they are a rich source of bioactive compounds. For example, sea buckthorn, a hardy deciduous shrub distributed especially in southwest, northeast, and northwest of China, contains a high yield of polyphenols in its leaves and fruit. The phytochemical components of three teas made from sea buckthorn leaves with different processing treatment were studied by high-performance liquid chromatography (HPLC), creating a chemophenetic fingerprint that was studied by multivariate statistical analysis (Wang et al.). A total of 48 compounds were identified, including four phenolic acids, 11 tannic acids, and 27 flavonoids. Due to the high yield and diversity of polyphenols, the sea buckthorn tea demonstrated inhibition of α-glucosidase activity and also conferred antioxidant effects *in vitro* (Wang et al.), suggesting that it is a good source of natural antioxidants. Oolong tea, another traditional Chinese tea made from partially fermented *Camellia sinensis* leaves, is especially popular in south China, and has also been proven to have potential health benefits. Li et al. found that 8 weeks of water supplementation of oolong tea, with dose of 318 mg polyphenols/kg body weight per day, could improve obesity by decreasing the levels of proinflammatory factors, and by alleviating metabolic challenges, such as fat accumulation, liver injury, glucose intolerance, and endotoxemia in high-fat diet (HFD)-fed mice. Oolong tea also regulated the expression of genes related to lipid metabolism and inflammation. Importantly, gut dysbiosis involving an increased *Firmicutes/Bacteroidetes* ratio and decreased diversity was resolved by oolong tea intervention in HFD-fed mice (Li et al.), indicating that it has significant potential as a dietary supplement for obesity and metabolic disorders.

To explore the biological effects of polyphenols further, a review by Chen et al. systematically reviewed the literature and commented on the potential network interaction mechanism of natural polyphenols in Chinese herbal medicine. The authors focused on the effects of metabolic homeostasis, immunity, and gut microbial regulation on obesity and diabetes. Indeed, metabolic disorders are strongly linked to immune imbalance, which may lead to chronic systemic inflammation. When pro-inflammatory cytokines are produced by immune cells it disrupts the metabolism of lipids and glucose, leading to insulin-resistance. Disturbance to gut microbiota increases gut permeability (leaky gut), leading to the entry of microbiota or endotoxic substances into intestinal tissues and systemic circulation. Ordinarily the metabolites of gut microbiota play an important role in regulation of immunometabolism (Chen et al.).

The profile of natural polyphenols and their yield in biota is affected by postharvest treatment and processing technology. Wei et al. revealed that 0.125 mM melatonin treatment of rambutan fruit inhibited electrolyte leakage, the accumulation of malondialdehyde and reactive oxygen species at the pericarp membrane, delaying the browning process or color loss during storage. Moreover, different processing treatment of sea buckthorn leaves were examined phytochemically, including fresh leaves, black tea produced by withering, rolling, fermentation and drying, and green tea produced by rolling, screening and drying process. Significant differences of polyphenol patterns occurred among the different processing tea (Wang et al.). The fermentation process makes the polyphenols susceptible to oxidation by enzymes, allowing catechins to be converted into theaflavins and thearubins, which confer the black tea fragrance and color.

In summary, the above-mentioned results demonstrate a valuable aspect to the composition, characterization, and bioactivities of natural polyphenols from fruit and traditional plants, in the context of preventing or controlling metabolic syndrome-associated diseases, especially obesity and diabetes. Polyphenols have a wide range of benefits and pharmacological effects. This reiterates that they serve as promising nutraceuticals or pharmaceuticals in health and food industries. Despite the evidence that has accumulated in relation to this important Research Topic, there are still many aspects that need to be clarified and improved. For example, low bioavailability of many polyphenols limits their application on food industry or the clinic. There are key techniques that may improve their bioavailability, such as formation of micelles, nanoparticles, liposomes, and phospholipid complexes. Converting polyphenols into ingredients that can be easily utilized through gut microbiota could be another way to promote the utilization of polyphenols (Chen et al.).

## Author contributions

All authors listed have made a substantial, direct, and intellectual contribution to the work and approved it for publication.
